# Neuroligins in neurodevelopmental conditions: how mouse models of *de novo* mutations can help us link synaptic function to social behavior

**DOI:** 10.1042/NS20210030

**Published:** 2022-05-10

**Authors:** Tobias T. Pohl, Hanna Hörnberg

**Affiliations:** Max Delbrück Center for Molecular Medicine, Robert-Rössle-Straße 10, Berlin 13125, Germany

**Keywords:** autism spectrum disorder, neurodevelopmental disorders, neuroligins, social behavior, synapses

## Abstract

Neurodevelopmental conditions (or neurodevelopmental disorders, NDDs) are highly heterogeneous with overlapping characteristics and shared genetic etiology. The large symptom variability and etiological heterogeneity have made it challenging to understand the biological mechanisms underpinning NDDs. To accommodate this individual variability, one approach is to move away from diagnostic criteria and focus on distinct dimensions with relevance to multiple NDDs. This domain approach is well suited to preclinical research, where genetically modified animal models can be used to link genetic variability to neurobiological mechanisms and behavioral traits. Genetic factors associated with NDDs can be grouped functionally into common biological pathways, with one prominent functional group being genes associated with the synapse. These include the neuroligins (Nlgns), a family of postsynaptic transmembrane proteins that are key modulators of synaptic function. Here, we review how research using Nlgn mouse models has provided insight into how synaptic proteins contribute to behavioral traits associated with NDDs. We focus on how mutations in different Nlgns affect social behaviors, as differences in social interaction and communication are a common feature of most NDDs. Importantly, mice carrying distinct mutations in Nlgns share some neurobiological and behavioral phenotypes with other synaptic gene mutations. Comparing the functional implications of mutations in multiple synaptic proteins is a first step towards identifying convergent neurobiological pathways in multiple brain regions and circuits.

## Introduction

Neurodevelopmental conditions (also referred to as neurodevelopmental disorders, hereafter shortened to NDDs) are a group of highly heterogeneous conditions that affect a range of behaviors in the cognitive and social domains [1,2]. These conditions, including autism spectrum disorder (ASD, hereafter referred to as autism [3]), attention deficit hyperactivity disorders (ADHDs), and intellectual disability, show overlapping symptoms, frequent co-diagnosis, and a shared genetic etiology [4,5]. In light of this high etiological and clinical heterogeneity, there has been a push towards a more transdiagnostic approach with a focus on distinct behavioral domains or physical symptoms rather than the primary diagnosis [1,6–8]. The Research Domain Criteria (RDoC) initiative, a framework launched by the National Institute of Mental Health (NIMH) in 2009 to tackle the heterogeneity and frequent comorbidities of psychiatric and neurodevelopmental conditions, identified six behavioral domains for cross-diagnosis research [6]. The proposed domains include systems for positive and negative valence, cognitive systems, systems for social processing, arousal/regulatory systems, and sensorimotor systems. Of these domains, altered social functioning is common in multiple neurodevelopmental and psychiatric conditions [9–12]. Differences in social interaction and communication are core diagnostic criteria for autism, and ADHD is associated with atypicalities in social functioning [13,14].

NDDs are heritable with a complex genetic underpinning consisting of both common and rare genetic variants [15–18]. In autism, the majority of heritability is made up of common genetic variants [16,17]. However, rare genetic variants contribute significantly to autism and other neurodevelopmental conditions, with more than 200 high confidence autism-associated genes currently identified ([17,19,20], see also https://gene.sfari.org/database/gene-scoring/). Genetic factors with a known function can be grouped functionally, and focusing on such common biological processes may be a way to reduce the genetic complexity and identify shared mechanisms between different genetic factors. One prominent functional group of genes linked to autism and other NDDs are those encoding proteins involved in synaptic formation and function [21]. These include synaptic cell-adhesion molecules that are crucial for the specialization and function of individual synapses, such as the neuroligins (Nlgns) and neurexins [22–24]. Out of these, Nlgns have received a lot of interest because of their fundamental roles at the synapse and high confidence association with autism and other NDDs.

Nlgns are a family of transmembrane cell adhesion molecules that are key regulators of synapse development and function [23,25,26]. Located at the postsynaptic site, Nlgns instruct synaptic differentiation through interactions with their presynaptic receptor neurexins [27–29]. Depending on which Nlgn and neurexin are expressed at the synapse, this connection specifies the formation of functional glutamatergic or GABAergic synapses. All Nlgns are considered strong candidate autism genes, and as a result, several mouse models with point mutations or knockdown (KD) of Nlgn proteins have been developed to study the pathophysiology of autism and related conditions. In this review, we discuss how Nlgn mouse models have informed our understanding of how synaptic alterations can drive changes at the circuit and behavioral levels. We focus on how altered Nlgn expression affects social behaviors, one of the core behavioral domains identified by the RDoC initiative, and describe how studying such rare genetic variants can help us understand the link between genetic variability at the synapse to changes in behavior.

## Nlgns, synaptic function, and neurodevelopmental conditions

**Figure 1:**
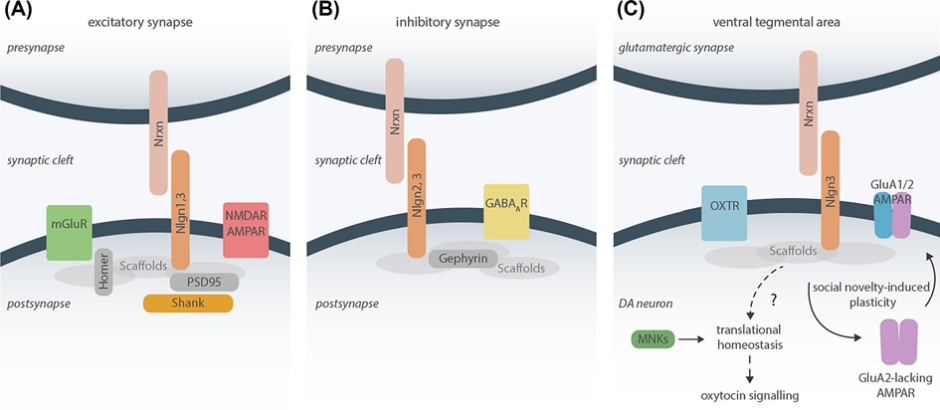
Nlgn complexes at the synapse Schematics of Nlgns at excitatory (**A**) and inhibitory (**B**) synapses. At excitatory synapses, Nlgns 1 and 3 interact with PSD95 and other scaffolding proteins important for the recruitment and maintenance of NMDAR and AMPAR to the synapse. At inhibitory synapses, Nlgns 2 and 3 interact with and recruit postsynaptic scaffolding proteins, including gephyrin, and inhibitory GABA_A_R to the synapse. (**C**) Besides the regulation of synaptic adhesion, Nlgns also affect other intracellular signaling pathways. For example, in VTA DA neurons, loss of *Nlgn3* disrupts translational homeostasis through as of yet unknown mechanisms [96]. KO of *Nlgn3* also disrupts oxytocin signaling, and restoring protein synthesis rate by treatment with an MNK inhibitor rescues oxytocin response and social recognition. Moreover, Nlgn3 affects synaptic plasticity in the VTA by regulating the social novelty-induced insertion of GluA2 lacking AMPARs at excitatory synapses on VTA DA neurons [95].

Synaptic function is one of the main cellular pathways targeted by rare *de novo* NDD and autism-associated genes, together with genes regulating translation and transcription [21]. The specialization and function of individual synapses are driven at least in part by a molecular code consisting of a combination of synaptic adhesion molecules, including the Nlgns and neurexins [22,24] (Figure 1). The human genome contains five Nlgns: *Nlgn1, 2, 3, 4X*, and *4Y*, with *Nlgn3* and *4X* localized on the X-chromosome, and *Nlgn4Y* (also referred to as *Nlgn5*) located on the Y-chromosome. Rodents only express four Nlgns (*Nlgn1*, *2*, *3*, and *4*), and with the exception of *Nlgn4* the protein sequences are highly conserved in mammals. The synaptic localization of Nlgns is specific, with Nlgn1 and Nlgn4X localized at excitatory synapses [30,31] (Figure 1A), Nlgn2 at inhibitory synapses [32] (Figure 1B), whereas Nlgn3 is expressed at both inhibitory and excitatory synapses [33] (Figure 1). Nlgns are essential for synaptic function and triple *Nlgn1–3* knockouts (KOs) are lethal despite showing normal number and structure of synapses [25].

Several mutations in the Nlgn genes have now been identified that are associated with autism and other neurodevelopmental and neuropsychiatric conditions [34–38]. The majority of these mutations map to the extracellular domain of the proteins [26]. Most of these mutations are found in the two X-linked Nlgn genes, *Nlgn4X* and *Nlgn3* [39]. *Nlgn4X* is most highly associated with autism, with multiple frameshift and missense mutations identified where most are thought to severely disrupt protein function [26]. However, *Nlgn3* has received the most attention, in part because of the high difference between the rodent and human *Nlgn4* sequence that has made any direct comparison of function more difficult than for the other Nlgns [40]. The association of Nlgns with autism was first reported in a Swedish family with two affected siblings, where one of the siblings had a *de novo* missense mutation in the coding region of *Nlgn3* and the other in *Nlgn4X* [35]. The *Nlgn3* mutation resulted in an R to C substitution at position 451 (R451C), causing misfolding of the protein and retention in the endoplasmic reticulum (ER). However, its binding ability to neurexin remained unaffected [41,42]. Therefore, the R451C substitution leads to a 90% reduction in protein levels within the forebrain, while the KO of *Nlgn3* completely abolishes the expression of the protein [43]. Other autism-associated Nlgn mutations are also suggested to cause ER retention and activation of ER stress pathways [26], suggesting that some phenotypes associated with these mutations may be caused by a gain-of-function rather than loss-of-function. Studying such effects of distinct mutations may reveal the biological basis for why mutations in the same gene contribute to multiple conditions or explain differences in symptom variability [44].

## The value of mouse models with *de novo* mutations in identifying pathogenic mechanisms

Genomic sequencing, and in particular whole-exome sequencing and whole-genome sequencing, have in the last decades identified an ever-increasing number of genes associated with autism and other NDDs. These range from ultrarare to common genetic variants that are also associated with behavioral traits in the general population [15,17,20,45,46]. Studying such a complex landscape of multiple genetic factors is not trivial, as it requires an extensive amount of data to draw conclusions. This is especially true for common genetic factors where a variation in any given single gene is unlikely to predict a specific outcome, and where the effect is dependent on a combination of other genetic and environmental factors. Although those challenges might be met with future research approaches applying mathematical models and computational methods to analyze multiple genetic factors [47], for now, preclinical research has primarily focused on mutations that disrupt or strongly affect the function of the encoded protein. These include genes associated with neurodevelopmental conditions with a known genetic cause that are frequently co-diagnosed with autism, such as Rett syndrome, Fragile X syndrome, and tuberous sclerosis [48]. As these monogenic disorders have overlapping symptoms, including difficulties or differences with social interactions and communication, integrating information from multiple single-gene conditions can be used to identify behavioral and biological pathways shared or distinct to a specific condition [1,8].

**Figure 2:**
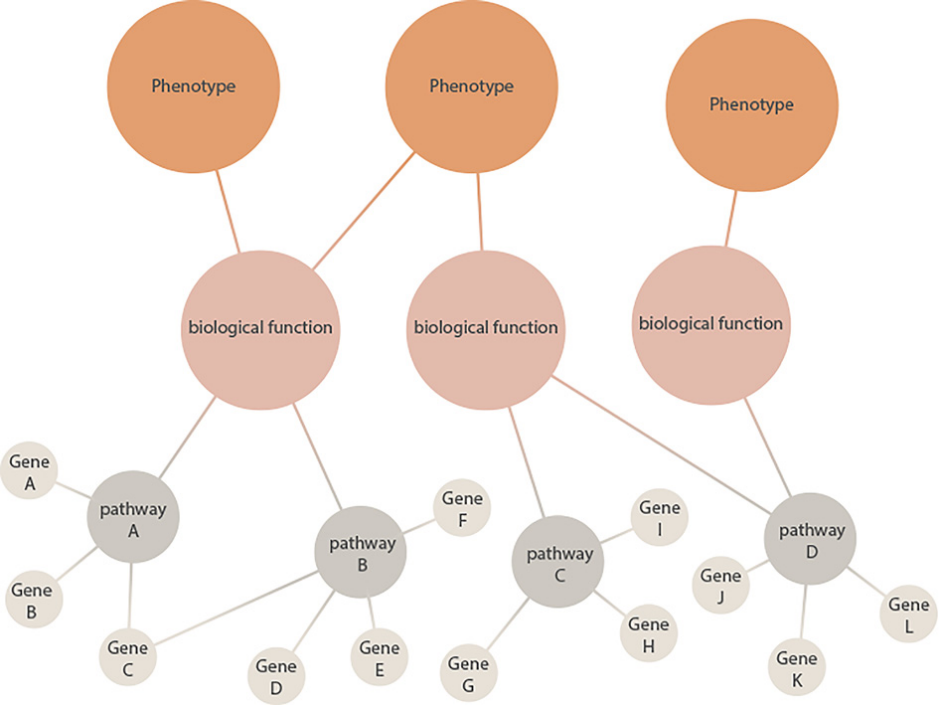
Shared pathways and biological functions governing behavioral domains Alterations in genes associated with NDDs may have an effect on shared or distinct biological pathways at the cellular level. These pathways, such as for example protein synthesis or synaptic signaling, can converge on higher level neuronal networks and biological functions, such as dopamine or oxytocin signaling. Such higher order biological function can, in turn, converge on to specific phenotypes or behavioral domains. As each behavioral domain is dependent on multiple biological functions, there can be several neurobiological mechanisms that would cause similar phenotypes. Analyzing the convergence of multiple genetic mutations on a pathway level and assessing their impact on biological functions can help to understand the neurobiological underpinnings of behavioral phenotypes.

For most genetic variants associated with NDDs there is limited knowledge at the clinical level. Here, animal models provide an essential platform to examine the link between genetic mutations and biological traits. Animal models are typically evaluated based on construct validity (the degree of etiological similarity to the human condition), face validity (the degree of phenotypic similarity between the animal model and the human condition), and predictive validity (similar response to treatment between the animal model and human condition). The large number of high-confidence genes associated with autism and other NDDs has led to the creation of multiple genetic models with strong construct validity. Although face validity can never be fully recapitulated for complex human conditions, genetic mouse models display several behavioral and neuroanatomical aspects of NDDs in a species-specific manner [49,50]. Therefore, examining the neurobiological consequences of rare *de novo* mutations such as the Nlgns in animal models can give important insight into how genetic variability affects the development and function of specific neuronal subtypes and circuits, and has the potential to reveal fundamental neurobiological principles of broader relevance to autism and NDDs without a known genetic cause. Given the rarity of each genetic mutation, it may be more useful to view animal models as a way to examine the biology underlying specific physiological or behavioral phenotypes rather than models for a specific diagnosis [8,51,52]. With this approach, the combination of multiple mouse models can be used to test the hypothesis that functionally grouped genetic mutations converge on to shared pathophysiological mechanisms (Figure 2). For example, the neurobiological and behavioral consequences of mutations in genes associated with the synapse can be compared with genes associated with cortical development or Wnt signaling to examine the relationship between gene function and phenotype [53,54]. Nlgns interact with multiple autism-associated proteins both directly and indirectly at the synapse (Figure 1). This includes the neurexins on the presynaptic site, where in particular neurexin1 is identified as a rare but significant genetic factor associated with multiple conditions, including autism and schizophrenia [55]. The cytoplasmic tail of Nlgns contains a PDZ-binding motif that directly interacts with the Shank family of postsynaptic scaffolding proteins [56,57], and all three Shank proteins are strongly associated with autism and other NDDs [58]. Together, this shows that a subset of autism-associated mutations may directly act in the same pathway at the synapse. Using animal models to study the Nlgns or other synaptic proteins in the context of such core synaptic pathways may therefore elucidate points of phenotypic convergence at multiple levels (Figure 2).

## Behavioral and molecular phenotypes in Nlgn animal models

Several Nlgn loss-of-function and mutation knockin mouse models have been developed in the last decades [26]. These models have been instrumental in elucidating Nlgns’ role at the synapse and exploring pathophysiological hypotheses such as altered excitatory/inhibitory balance [23,26]. We will focus here on Nlgns’ role in circuit-specific function underlying social behaviors, one of the core behavioral domains that overlap between multiple neurodevelopmental and psychiatric conditions [6,59,60].

### Social behaviors in mouse models

The behavioral domain approach is well suited to animal models, where genetic- or environment-based models can be used in combination with circuit- and cell type-specific manipulation. Rodents are innately social animals with a wide array of social behaviors that can be modulated by previous experience or genetic background [61–63]. Mice are motivated to seek out social interactions and have a broad spectrum of prosocial behaviors, including helping behavior and social transmission of emotions, suggesting mice are capable of complex forms of social cognition [64–69]. Although species-specific, strain-specific, and individual differences in sociability and social strategies need to be taken into account [70], evidence supports a degree of conservation in the neural circuits and cellular mechanisms that regulates social behaviors across mammalian species [71,72,59,73]

There is a vast array of social behavioral tests in rodents developed to assess different aspects of social behaviors such as social preference, anxiety, reward, and memory, and these tests are routinely used to understand the circuitry and cellular pathways involved in altered social states [74]. More recently, non-invasive monitoring of reciprocal social behaviors in group-housed mice has been developed to provide a more naturalistic assessment of complex social phenotypes [75–78]. Capturing naturalistic behaviors in the home cage environment facilitates the detection of behavioral patterns that are otherwise not possible to detect under standard experimental settings, such as self-selected reciprocal social behaviors. Animals behaving in naturalistic environments and group settings display a diverse behavioral range that can be detected by unsupervised behavioral analyses. This can facilitate distinguishing between behavioral pattern differences, rather than *ad hoc* defined and potentially human experimenter-biased behavioral classifications.

### Role of Nlgns in social behaviors

Given their broad expression patterns and essential roles in synaptic specificity and function, it is perhaps not surprising that mutations in all Nlgns, either loss- or gain-of-function, affect social behaviors in rodent models. The magnitude and nature of affected social behaviors depend on the specific Nlgn and type of mutation. As such, examining the neurobiological and behavioral consequences of these mutations can help determine the role of a particular Nlgn in the development and function of the social brain. A summary of Nlgn animal models and their social phenotypes are outlined in Table 1.

**Table 1:** Effect of Nlgn alterations on social behaviors

Gene	Species	Age	Sex	Alteration	Molecular/Physiology	Behavior	References
*Nlgn1*	Mouse	adult	male, female	KO	**DS:** NMDA/AMPA ratio ↓; **CA1**: LTP ↓	social interaction ↓, social preference ↔, social novelty ↔, social recognition ↔	[79]
	Mouse	adult	male	Pro89Leu		social preference ↓, social dominance ↓, courtship vocalization ↔	[36]
	Mouse	pup	male, female	Pro89Leu		isolation USV ↔	[36]
*Nlgn2*	Mouse	adult or adolescent	male	OE	**mPFC:** E/I ratio ↓, mIPSC frequency ↑, inhibitory synaptic density ↑	social interaction ↓, social preference ↓	[85]
	Mouse	juvenile, adult		KO		isolation USV ↓, adult social preference ↔	[81]
	Rat	adult	male	dHipp OE	**dHipp:** Increased GAD65 expression	social preference ↔, social novelty ↔, aggression ↓	[89]
	Rat	adult	male	Hipp Nlgn2 peptide infusion	**Hipp:** ↓ Nlgn2 expression linked to stress susceptibility	aggression ↑, social preference ↓	[87]
	Mouse	adult	male	Hipp OE or KD	**dvHipp:** ↑ Nlgn2 linked to ELS susceptibility	**Hipp KD:** social preference ↔, social novelty ↔**Hipp OE:** social preference ↔, social novelty ↓, aggression ↑	[86]
	Mouse	early adulthood	male	cNlgn2 KD in NAc D1 or D2	**NAc:** Nlgn2 ↓ in D1 MSNs linked to stress susceptibility	**NAc D1 KD after subthreshold social defeat stress:** social avoidance ↑, defensive behavior ↔, social dominance ↓ **NAc D2 KD after subthreshold social defeat stress:** social avoidance ↔, defensive behavior ↑, social dominance ↑	[83]
	Mouse	adult	male, female	KO	**Thalamocortical circuit:** impaired GABAergic transmission	social preference ↔, social novelty ↓, social recognition ↔	[80]
*Nlgn3*	Mouse	adult	male	R451C	**CA1/CA2/SSC:** VGAT density ↑; **SSC:** inhibitory synaptic strength ↑	social preference ↓, social novelty ↓, social recognition ↔	[43]
	Mouse	pup, juvenile, adult	male, female	R451C		**male pups:** isolation USV ↑ **female pups:** isolation USV ↔ **juveniles:** sociability ↔ **adults:** social preference ↔, social novelty ↔	[92]
	Mouse	adult	male	KO	Reduced total brain volume	social interaction ↔, social preference ↔, social novelty ↓, courtship vocalization ↓	[98]
	Mouse	adolescent, adult	male	R451C	**CA1/Hipp:** NMDA/AMPA ratio ↑, LTP ↑, dendritic branching ↑, spine density ↔, excitatory transmission ↑, NR2B-containing NMDA receptors ↑; SSC: inhibitory transmission ↑	social preference ↔, social novelty ↓	[93]
	Mouse	adult	male	KO		courtship vocalization ↓	[99]
	Mouse	adult	male	R451C	**mPFC:** c-Fos following social novelty ↓, low γ oscillations ↓, impaired local phase–amplitude coupling and functional encoding during social interaction	social preference ↔, social novelty ↓	[91]
	Mouse	adult	male	R451C		social preference ↔	[94]
	Mouse	adolescent, adult	male	KO, VTA::DA^NL3KD^	**VTA:** GluA2-lacking AMPAR ↑ at inputs to DA neurons	**adult KO:** social interaction ↓, recognition ↓ **adolescent KO:** social reward ↓ **adult VTA::DA^NL3KD^:** social interaction ↓, social recognition ↓ **adolescent VTA::DA^NL3KD^:** social reward ↓ **adult VTA::DA^NL3KD-adult KD^:** social recognition ↓	[120]
	Mouse	adult	male	KO	**CA2:** altered θ and γ power, spike-triggered average ↓, GABA release from CCK+ interneurons ↓; **CA3**: altered θ and γ power	social preference ↓, social novelty ↓	[97]
	Mouse	juvenile	male	KO	**VTA:** altered translational regulation and OT response in DA neurons	social recognition ↓	[96]
*Nlgn4*	Mouse	adult	male	KO	Reduced total brain volume	social interaction ↓, social preference ↓, social novelty ↓, aggression ↓, courtship vocalization ↓	[107]
	Mouse	pups, juvenile, adult	male, female	KO		**juveniles:** social interaction ↔ **adults:** social preference and social novelty ↔ **pups:** isolation USV ↔	[109]
	Mouse	adult	male, female	KO		**females:** social interaction ↓, social preference ↔, social novelty ↓, USV to anesthetized female ↓ **males:** social interaction ↓, social preference ↓, social novelty ↔, courtship vocalization ↓, aggression ↓	[106]
	Mouse	pups, juvenile	male, female	KO		**pups:** isolation USV latency ↓ in females **juveniles:** isolation USV ↓ with stronger phenotype in females	[108]

**Reported alterations:** ↑ increase, ↓ decrease, ↔ no change.

Abbreviations: CA1/CA2, CA1/CA2 region of the hippocampus; DA, dopamine; dHipp, dorsal hippocampus; DS, dorsal striatum; dvHipp, dorsoventral hippocampus; ELS, early-life stress; E/I, excitation/inhibition; Hipp, hippocampus; LTP, long-term potentiation; mPFC, medial prefrontal cortex; MSN, medium spiny neuron; NAc, nucleus accumbens; OE, overexpression; OT, oxytocin; SSC, somatosensory cortex; USV, ultrasonic vocalization; VTA, ventral tegmental area.

**Behavior:** Social preference is defined as a preference for a social versus an inanimate object in a three-chamber test or similar. Social novelty is a preference for a novel versus familiar mouse in a three-chamber test or similar. Social recognition is defined as a preference for social novelty when there is a memory component involved. Social interaction refers to direct interaction between two freely moving mice.

Nlgn1 is located on excitatory synapses throughout the brain. Global KO of the *Nlgn1* gene results in modest alterations in social behaviors, with reduced interaction with a caged adult novel mouse, but no change in social preference or social novelty in the three-chamber paradigm (although it should be noted that in the same study wildtype mice also failed to show a statistically significant social preference, making this result harder to interpret) [79]. Similarly, knockin of the autism-associated *Nlgn1* mutation Pro89Leu causes a small but significant decrease in social preference and social dominance [36]. In the *Nlgn1* KO mouse, this change in social behavior was coupled with reduced NMDA/AMPA ratio in the dorsal striatum (DS) and decreased long-term potentiation (LTP) in the CA1 region of the hippocampus (Hipp) [79]. However, whether these changes are causally linked to altered social behaviors was not determined.

In contrast with *Nlgn1*, mice lacking the inhibitory *Nlgn2* do not show any difference in social preference in the three-chamber test, but display reduced preference for social novelty in the same test [80,81]. Somewhat surprisingly, social novelty preference was intact in a habituation/dishabituation test in the same study. The altered social preference was linked to impaired GABAergic transmission in thalamocortical circuitry that also caused absence seizures. Pharmacological treatment with an anti-seizure drug restored both GABAergic transmission and social novelty preference, suggesting that both of these phenotypes may share altered excitatory/inhibitory balance as an underlying cause [80]. *Nlgn2* has been implicated in negative social processing and aggressive behavior, with several publications showing a link between altered *Nlgn2* expression and aggression-related behaviors, anxiety, and altered social behaviors [82–87]. Hines et al. (2008) show that Nlgn2 overexpression (OE) alters the excitation/inhibition (E/I) ratio in the medial prefrontal cortex (mPFC) and reduces social preference. Although the exact circuitry was not investigated, it is intriguing to speculate whether this E/I ratio alteration affects the mPFC–BLA circuitry which is involved in regulating social preference [88]. In mice, early-life stress (ELS) causes an increase in *Nlgn2* expression in both dorsal and ventral hippocampus, and KD of *Nlgn2* during adulthood rescues ELS-induced social phenotypes. The same study showed that OE of *Nlgn2* in the hippocampus mimics the effect of ELS by reducing social recognition and increasing aggressive behavior, whereas KD did not affect social behaviors [86]. Interestingly, in rats, OE of *Nlgn2* in hippocampus reduces aggressive behavior while leaving social interaction and social novelty preference intact, suggesting that although Nlgn2 levels in hippocampus are linked to aggression in both species, the relationship between expression level and aggression differ between mice and rats [89]. In adult mice, chronic stress appears to cause an opposite effect on Nlgn2 expression compared with ELS, with reduced expression levels in both hippocampus and nucleus accumbens (NAc) associated with stress susceptibility [83,87]. In the NAc, Nlgn2 plays a cell type-specific role in stress susceptibility with stress causing a reduction in Nlgn2 in D1-positive, but not D2-positive medium spiny neurons (MSNs) [83]. This promotes social avoidance, probably via a decrease in inhibitory synapses [84]. A similar decrease in Nlgn2 expression was found in the NAc in postmortem samples of depressed patients, suggesting that regulation of interneuron synapses via Nlgn2 expression could be a key regulator of stress response in multiple brain regions and developmental timepoints. A recent study demonstrated a link among γ-protocadherins, Nlgn2 expression, and social and anxiety-like behaviors, suggesting γ-protocadherin as possible negative regulators of Nlgn2-driven inhibitory synapse formation and function [90].

Both *Nlgn3* loss-of-function and the *Nlgn3* autism-associated *R451C* mutation affect social behaviors in mice, although with some differences. *Nlgn3 R451C* mutations lead to heterogeneous and strain-specific effects on sociality [43,91–94], whereas *Nlgn3* loss-of-function more consistently affects social interaction, social reward, and social recognition in multiple tests [95–99]. Part of these differences between the *R451C* mutation and global KO mice may be driven by a different effect on Nlgn3 function depending on its interaction with either neurexins or protein tyrosine phosphatase δ (PTPδ), where the R451C mutation selectively impairs PTPδ interaction [100]. In the *Nlgn3^KO^* mice, altered social behaviors are associated with impaired novelty-dependent neural plasticity on to dopaminergic neurons in the ventral tegmental area (VTA) [95,96]. Social interactions normally strengthen excitatory synaptic inputs on to dopaminergic VTA neurons, but this synaptic potentiation is absent from mice lacking *Nlgn3* [95]. This effect is specific to *Nlgn3’s* expression at dopaminergic VTA neurons, as dopamine (DA)-specific loss of *Nlgn3* is sufficient to cause the phenotype [95]. Loss of *Nlgn3* also impairs oxytocin (OT) signaling of dopaminergic VTA neurons. OT neurons in the paraventricular nucleus project directly on to dopaminergic neurons in the VTA, and the release of OT on to dopaminergic VTA neurons modulates several aspects of social behaviors [65,96,101,102]. In mice lacking *Nlgn3*, the loss of OT response is not caused by a direct change in oxytocinergic signaling, as the expression of OT and its receptor was unaltered in the *Nlgn3^KO^* mice. Instead, the reduced OT response is linked to altered activity-dependent protein synthesis in dopaminergic VTA neurons [96] (Figure 1C). OT signaling is necessary for both social novelty and social reward in mice [65,96,103], and altered social novelty response has been linked to OT in both dopaminergic VTA neurons and the CA2 region of the hippocampus [104,105]. Interestingly, loss of *Nlgn3* causes altered network dynamics in the CA2 [97], opening the possibility that *Nlgn3* may modulate OT-dependent signaling in multiple social brain regions.

KO of the other X-linked Nlgn, *Nlgn4*, also affects social behavior, with adult male and female mice showing reduced social interaction and social communication [106–108], although it should be noted that another study failed to replicate these findings [109].

Mutations in Nlgns also affect social communication. During development, isolated *Nlgn2^KO^* pups elicit less ultrasonic vocalizations (USVs) than their wildtype counterparts [81]. One explanation for reduced pup vocalizations might be differences in the individuals’ state anxiety, which is modulated by maternal care. However, anxiety is not affected in adult individuals, which might be indicative of a general communication deficit as vocalization phenotypes are found in multiple neuroligin KO and knockin models [92,98,99,107]. For example, altered vocalization during development is also present in mice carrying the *Nlgn3 R451C^KI^* mutation, where reduced USVs are observed in male pups [92]. *Nlgn4*^*KO*^ pups and juvenile mice also display altered USV, with a more pronounced phenotype in females [108]. In adulthood, reduced vocalization during courtship is found in both *Nlgn3* and *Nlgn4* KO male mice [98,99,107], whereas loss of *Nlgn4* also reduces USV in adult females [110]. Interestingly, despite their different expression profile, both *Nlgn3* and *Nlgn4* loss-of-function have similar effects on USV, with a reduced number of calls and increased latency to the first call [107,110]. USVs are modified by altered synaptic transmission or neuronal activation in specific brain regions. The periaqueductal gray (PAG) is responsible for gating USVs and is innervated by the preoptic area (POA) and the central-medial amygdala (CeM) [111]. Whereas activation of GABAergic CeM-PAG projecting neurons blocks male social vocalizations towards females, activation of GABAergic POA-PAG projections leads to vocalizations in the absence of social stimuli [111,112]. It is intriguing to speculate that changes in Nlgn-dependent synaptic communication, including differential gene expression, protein translation, and neuropeptide signaling, might affect neural inputs into the PAG deriving from the POA and the CeM. Another possibility is that mutations in Nlgns affect vocalization indirectly as a consequence of altered social processing or social motivation. Notably, the CeM receives input from brain regions involved in the mesolimbic reward pathway, including the amygdala and VTA [113,114]. As *Nlgn3* is necessary for DA neuron-dependent social behaviors including social novelty perception and social reward [95], it is possible that loss of *Nlgn3* may affect vocalization indirectly via this circuit.

In summary, multiple studies support a role for Nlgns in regulating several different aspects of social behavior with the effect at least partially dependent on the specific Nlgn involved. Perhaps not surprising given their expression pattern, Nlgn2 appears to affect sociability via regulation of inhibitory synapse transmission, while Nlgn3 affect social reward and social novelty recognition via AMPA-receptor plasticity and OT response at dopaminergic VTA neurons [80,83,86,95,95]. Most of the studies examining Nlgns’ role in social behaviors are performed on brain-wide KO or manipulation of the protein expression. However, as altered Nlgn expression affects a range of neurobiological and behavioral domains that may directly or indirectly impact the formation and function of social brain circuits, results from full KO animals may be harder to interpret. More research is needed to gain information on the local versus global action of Nlgns on specific aspects of social interaction and social cognition, and how different Nlgns can intersect to influence the same behavior at the circuit level [115].

## Identifying convergent mechanisms

Given their crucial roles as synaptic organizers and wide expression patterns, it is not surprising that mutation in Nlgn genes affects a multitude of mechanisms, from cell adhesion to intracellular signaling processes. Examining their synaptic function and how they intersect with other genes associated with NDDs can help identify mechanistic points of convergence at the cellular and molecular levels. Nlgns interact directly with other autism-associated genes, such as the neurexins and shank proteins, and comparing pathophysiologies that are shared versus distinct across synaptic proteins could improve our understanding of which therapeutic approaches could be helpful. For example, multiple autism-associated synaptic proteins, including Nlgn3, Shank3, and FMRP, affect group-I or group-5 metabotropic glutamate receptors (mGluRs) [116,117,60]. mGluRs are G protein-coupled receptors important for synaptic plasticity. Contrasting the expression profiles and functions of synaptic proteins that affect mGluRs, and determining how this maps on to developmental processes or behavioral domains, may enable a better understanding of which types of pathophysiologies’ drugs targeting mGluRs could be helpful.

Convergent mechanisms can also be found at the level of neurotransmitters or neuromodulators despite different primary cellular mechanisms. Changes in the neuropeptide OT have been suggested as a possible treatment approach for social symptoms in autism and other NDDs, primarily for its role as a social modulator. OT is thought to increase the salience of social cues by increasing the signal-to-noise ratio of social cues within the social behavioral network [118], thus elevating social-related learning and perception in social contexts. OT-induced activation of dopaminergic neurons in the VTA is necessary for multiple aspects of social behavior [65,96,102,119], and multiple studies in rodents have linked changes in social behaviors with the oxytocinergic and dopaminergic systems [67,96,119–123]. Altered OT signaling and dopaminergic neuron plasticity are found in several mouse models with mutations in genes associated with the synapse. This includes *Shank3*, *Ctntp2*, and *Nlgn3* KO mice, although the mechanism of action differs [96,119,122,124]. In *Shank3* and *Ctntp2* KO mice, there is a general reduction in OT-positive neurons in the paraventricular nucleus caused by an altered gut microbiome [119,125], whereas mice lacking *Nlgn3* have no changes in OT-positive neurons or receptor expression. Instead, the reduced OT response is caused by altered protein synthesis in dopaminergic VTA neurons [96]. These differences in mechanism may give some explanation as to why treatment with OT has yielded mixed results.

The dynamic changes in synaptic strength that underlie learning and memory require remodulation of the synaptic proteome via *de novo* protein synthesis and protein degradation [126,127]. Several autism and NDD-associated genes directly affect protein synthesis or degradation [21], and dysregulation in ERK and mTOR pathways has been found in autistic children [128]. Because of this, dysregulation in synaptic proteome homeostasis has been proposed as a possible unifying mechanism underlying some of the traits and symptoms associated with autism and other NDDs [21,129]. Recently, it has been shown that mutations in several synaptic genes, including Nlgns, also affect protein synthesis [96,130–133]. *Nlgn3^KO^* mice have altered activity-dependent translational regulation in dopaminergic VTA neurons coupled with a general increase in ribosomal proteins in the VTA [96]. Interestingly, altered expression of ribosomal proteins has also been found in a mouse model lacking *Shank3* as well as in human cell lines and brain tissue from autistic individuals, suggesting that altered ribosomal biogenesis may be a more widespread feature in autism [130,134–136]. In *Nlgn3^KO^* mice, the increase in protein synthesis is likely directly linked to altered social behavior as treatment with an MAP-kinase interacting kinase (MNK) inhibitor could restore not only protein synthesis and ribosomal protein expression but also social behavior [96]. MNK activity modulates the translational activity of the Fragile-X-disorder protein Fmrp [137], and inhibition of MNKs or their downstream target eIF4E restores protein synthesis and social behaviors in a Fragile X mouse model [138]. Interestingly, KO of the eIF4E repressor 4E-BP2 or microglia-specific overexpression of eIF4E increases the expression of Nlgns while decreasing social interaction [139,140]. Together, this suggests a convergence between multiple synaptic autism-associated genes and regulation of translation, pointing to a possible shared core plasticity element among multiple seemingly unrelated genetic factors.

## Conclusion and future perspective

The use of animal models to study the functional consequence of rare *de novo* mutations has been instrumental in providing mechanistic links among gene function, neural circuitry, and behavior. Neural networks in mice share multiple properties with humans, and most existing medications for psychiatric conditions are at least partially effective in mice, supporting the notion of evolutionary conserved neurobiological mechanisms relevant to pathophysiological outcomes [49,141]. Therefore, although animal models can never recapitulate the full complexity of a human condition, comparing and contrasting multiple models provide a pathway towards understanding the biology underlying specific traits or symptoms associated with NDDs and autism. To this end, examining mutations in different Nlgn proteins can identify points of convergence between synaptic proteins that differentially affect inhibitory or excitatory signaling. The points of convergence range from behavioral phenotypes co-observed between different mutations, to shared biological pathways affecting specific neuronal circuits. Furthermore, comparing the phenotypes of animal models with mutations in synaptic proteins that interact with Nlgns can highlight shared molecular pathways and link them to behavioral phenotypes. In this way, it may be possible to reduce the high etiological heterogeneity to a smaller subset of common neurobiological mechanisms. Future studies will benefit from more spatial and temporal resolution of molecular changes in the Nlgn proteins, and linking these changes to cellular and circuit dysfunction that can be compared and validated in humans. On the behavioral side, future studies will greatly benefit from advances in semi-naturalistic behavioral phenotyping and machine learning to identify not just relevant behavioral domains but also individual differences across developmental trajectories.

Another important source of heterogeneity in autism and other NDDs comes from individual variability. Even NDDs with a monogenetic cause can display large phenotypical and neurobiological variability, demonstrating that individual consideration is crucial. Here, animal models can be used to understand how multiple genetic and environmental factors interact to modulate behavioral phenotypes in animals with the same underlying genetic mutation. Even genetically almost identical mice display individual behavioral characteristics, including differences in social behaviors [142,143]. Understanding how individual phenotypical heterogeneity is encoded on the cellular and molecular level will help explain why certain types of treatment are only effective for some individuals despite similar genetic mutations or similar underlying symptoms. Moreover, detecting shared molecular pathways and biological functions underlying behavioral phenotypes across variable genetic backgrounds, both within and between species, can present evidence for evolutionary conserved neurobiological mechanisms of certain behavioral responses. Nlgns are conserved across multiple species from *Caenorhabditis elegans* to humans, and cross-species comparison of neurobiological mechanisms may aid in translating preclinical research to human application [30,144–147].

In summary, although some symptomatic features are likely to be unique to humans, studying rare genetic *de novo* mutations such as the Nlgns in animal models is essential to identify cellular and circuit mechanisms underlying specific symptoms. Combining information from multiple models, including Nlgn mutations in patient-derived stem cells or organoids [30], will help to gain a broader understanding of the neurobiological function of Nlgns and how this relates to the pathophysiology of neurodevelopmental conditions.

## Data Availability

Data sharing is not applicable for the present paper.

## Abbreviations

ADHD: attention deficit hyperactivity disorder
AMPA: α-amino-3-hydroxy-5-methyl-4-isoxazolepropionic acid
CeM: central-medial amygdala
DA: dopamine
DS: dorsal striatum
E/I: excitation/inhibition
eIF4E: eukaryotic translation initiation factor 4E
ELS: early-life stress
ER: endoplasmic reticulum
Hipp: hippocampus
KD: knockdown
KO: knockout
mGluR: metabotropic glutamate receptor
MNK: MAP-kinase interacting kinase
mPFC: medial prefrontal cortex
NAc: nucleus accumbens
NDD: neurodevelopmental disorder
Nlgn: neuroligin
NMDA: N-methyl-D-aspartate
OE: overexpression
OT: oxytocin
PAG: periaqueductal gray
POA: preoptic area
RDoC: Research Domain Criteria
USV: ultrasonic vocalization
VTA: ventral tegmental area
4E-EB2: eukaryotic Translation Initiation Factor 4E Binding Protein 2
